# CTA-based risk assessment of the carotid variant of Eagle syndrome: development and internal validation of a nomogram

**DOI:** 10.3389/fneur.2025.1699139

**Published:** 2025-10-30

**Authors:** Yudan Liu, Shengtao Sun, Min Sun, Zhiwei Wang, Jianguo Liu, Xiaokun Qi, Chenjing Sun

**Affiliations:** ^1^Senior Department of Neurology, The First Medical Center of PLA General Hospital, Beijing, China; ^2^Department of Stomatology, The Sixth Medical Center of PLA General Hospital, Beijing, China; ^3^Department of Ophthalmology, The Sixth Medical Center of PLA General Hospital, Beijing, China; ^4^The Second Affiliated Hospital of Anhui Medical University, Hefei, China

**Keywords:** Eagle syndrome, vascular Eagle syndrome, ischemic stroke, carotid artery dissection, carotid artery stenosis, nomogram

## Abstract

**Background:**

Eagle syndrome (ES) is uncommon; its carotid variant [ES–CA, sometimes termed vascular Eagle syndrome (VES)] can produce internal carotid artery (ICA) dissection or stenosis and ischemic stroke, yet is frequently underrecognized. This study leveraged large-sample computed tomography angiography (CTA) to quantify structural determinants of styloid–ICA contact and to develop and internally validate a nomogram for early risk stratification.

**Methods:**

We retrospectively included 414 consecutive head–neck CTA examinations (January 2023–March 2025). Volume rendering (VR) and maximum intensity projection (MIP) were used to delineate styloid–vessel relationships and to measure styloid process length (SPL), anterior tilt angle (FTA), and medial inclination angle (IA). Univariable/multivariable logistic regression identified correlates of ICA contact; receiver operating characteristic (ROC) analyses compared alternative SPL metrics (ipsilateral, bilateral mean, bilateral maximum) to select the optimal predictor. A nomogram incorporating significant predictors underwent 1,000-bootstrap internal validation with assessment of discrimination, calibration, and decision-curve analysis (DCA).

**Results:**

ICA contact was present in 110/414 (26.6%). Men had longer styloids and larger FTAs than women (both *p* < 0.001), but smaller IAs (left: 19.00° vs. 21.00°, *p* < 0.001; right: 22.00° vs. 23.00°, *p* = 0.010). Female sex independently predicted ICA contact (OR = 3.838, *p* < 0.001), and SPL on both sides was an independent risk factor (left OR = 1.063; right OR = 1.085; both *p* < 0.05). Sex-stratified models revealed laterality: in men, right-sided SPL (OR = 1.101, *p* = 0.006) was decisive; in women, left-sided SPL (OR = 1.092, *p* = 0.050) was decisive. Among SPL metrics, the bilateral maximum (SPL-max) performed best for predicting contact (overall AUC = 0.731; men = 0.787; women = 0.733) with sex-specific cut-offs of 30.20 mm (men) and 26.75 mm (women). The nomogram combining SPL-max, sex, and age showed good performance (AUC = 0.779; calibration slope = 0.96) and yielded positive net benefit on DCA across 1–65% threshold probabilities.

**Conclusion:**

Risk of ES–CA–related ICA contact was unrelated to age or angular parameters. Styloid length and sex were the principal structural risk factors, with right-sided predominance in men and left-sided predominance in women, suggesting sex–side interaction. SPL-max was the optimal predictor, with a 3.45-mm lower cut-off in women, and the internally validated nomogram demonstrated clinical utility for early, imaging-based screening.

## 1 Introduction

Eagle syndrome (ES), also termed styloid process syndrome (SPS), is a relatively rare disorder of the craniocervical junction, with a reported prevalence of 4–28% (1, 2), and women are affected approximately three times as often as men (3). Although early case descriptions can be traced back to the 19th century, Eagle’s papers in 1937–1938 systematized and popularized the entity rather than constituting the very first description (4, 5), and the condition is therefore commonly known as Eagle syndrome. The typical pathophysiology involves mechanical irritation or compression of adjacent structures by an elongated styloid process (ESP) and/or a calcified stylohyoid ligament, producing cervical pain, globus sensation, or referred otalgia; patients often present not only to otolaryngologists but also to maxillofacial surgeons and dentists (6).

On imaging, the styloid process length (SPL) is generally 2.5–3.0 cm, and >3.0 cm is commonly considered elongated in prior literature (7); moreover, lengths >4.0 cm have often been associated with pain in previous studies (8). Angular abnormalities are typically characterized by the medial inclination angle (IA) and anterior tilt angle (FTA); IA >15° and FTA >30° are frequently used thresholds for abnormality (9). Theoretically, a larger IA increases the likelihood of contact with the internal carotid artery (ICA), whereas a larger FTA may irritate the tonsillar fossa and nearby neurovascular structures, resulting in pharyngeal paresthesia or pain (10). However, the relative contributions of length and angular parameters to disease expression remain uncertain, and the correspondence between radiographic “abnormalities” and clinical symptoms shows substantial interindividual variability.

Despite its infrequent clinical presentation and limited awareness among neurologists, this uncertainty may permit ICA compression to progress to carotid artery dissection before diagnosis in a subset of patients with ES (11). Based on the involved structure and clinical features, ES is generally recognized in three variants: (1) the “classical” type associated with otolaryngologic symptoms; (2) the carotid variant, originally described by Eagle, in which the styloid process compresses the carotid artery and may provoke neck pain, transient ischemic attacks (TIA), or ischemic stroke; and (3) the jugular variant, in which an abnormal styloid process compresses the internal jugular vein, leading to impaired venous outflow or intracranial hypertension (12). The present study focuses on the carotid variant of Eagle syndrome (ES–CA), i.e., mechanical compression of the ICA by the styloid process. In ES–CA, direct contact and/or indentation of the ICA can precipitate carotid dissection or stenosis and subsequent cerebral ischemia; this variant is estimated to account for ~4–10% of ES cases (10, 13).

Because presenting symptoms are often nonspecific, early recognition of ES–CA is challenging. Some patients report only headache, dizziness, or tinnitus without focal neurologic signs, whereas others experience ischemia in the ICA/MCA territories or stroke with unrecognized structural risk before surgery (13). Accordingly, elongation alone is not sufficient for diagnosis; vascular wall vulnerability, local inflammatory changes, and repetitive mechanical friction may act in concert to facilitate carotid dissection (14, 15). Given the potential stroke risk, defining the anatomic relationship between the styloid process and the ICA and quantifying risk thresholds are clinically important. In this context, we target the imaging-defined structural state of “styloid–ICA contact” in ES–CA, use large-sample CTA-based three-dimensional measurements to quantify the impact of SPL and angular parameters on ICA contact, and develop a nomogram with sex-stratified cut-offs to provide a quantitative tool for identifying individuals at elevated risk.

With respect to assessment, compared with conventional anteroposterior and lateral radiographs, computed tomography angiography (CTA) with three-dimensional reconstruction—specifically volume rendering (VR) and maximum intensity projection (MIP)—more accurately delineates the three-dimensional spatial relationship between the styloid process and the ICA (16). VR preserves anatomic landmarks and enables multiplanar visualization to track the styloid in relation to adjacent vessels, while MIP highlights high-attenuation, contrast-enhanced vessels and helps judge luminal deformation or dissection. When indicated, transcranial Doppler (TCD) can be used to evaluate ICA flow changes during neck rotation and to assess the feasibility and potential benefit of styloidectomy (10). In sum, the present study adopts styloid–ICA contact as the primary structural endpoint, systematically analyzes anatomic correlates, and constructs an early prediction model to support imaging-guided decision-making in ES–CA.

## 2 Methods

### 2.1 Study design and participants

This was a single-center retrospective observational study including 414 consecutive outpatients or inpatients who underwent head–neck computed tomography angiography (CTA) in the Department of Neurology between January 1, 2023 and March 30, 2025. Among them, six cases were diagnosed with the carotid variant of Eagle syndrome (ES–CA) and exhibited internal carotid artery (ICA) dissection or stenosis. No participant had received any intervention directly related to vascular Eagle syndrome (VES) prior to imaging. The study focused on structural imaging parameters and their association with ICA contact; treatment effects were not assessed.

### 2.2 Imaging acquisition and three-dimensional reconstruction

CTA datasets were post-processed using volume rendering (VR) and maximum intensity projection (MIP) to delineate the three-dimensional spatial relationship between the styloid process and adjacent structures, particularly the ICA. The styloid process length (SPL) was defined as the linear distance from the inferior margin of the external acoustic meatus to the styloid tip and was measured separately on both sides. For each patient, we additionally computed the bilateral mean SPL (mean of left and right) and the bilateral maximum SPL (max of left or right). Angular parameters included the medial inclination angle (IA) and the anterior tilt angle (FTA), which were measured on standardized planes.

### 2.3 Ascertainment of ICA contact status

To objectively characterize the styloid–ICA relationship, we prespecified two imaging groups based on VR/MIP reconstructions: (1) Contact group—direct contact between the styloid process and the ipsilateral ICA, with or without luminal indentation; and (2) Non-contact group—an evident anatomic gap without direct contact or deformation. All images were independently reviewed in a blinded fashion (clinical data masked) by two board-certified radiologists at the associate chief level or above; disagreements were adjudicated by a third senior radiologist. Interrater agreement for ICA-contact classification was good (Kappa = 0.82).

### 2.4 Statistical analysis

All analyses were performed in R (version 4.1.0). Continuous variables were first assessed with the Shapiro–Wilk test. Normally distributed data are reported as mean ± standard deviation; non-normally distributed data as median and interquartile range (IQR). Categorical variables are presented as counts and percentages (*n*, %). Between-group comparisons used the Mann–Whitney *U* test for non-normal data and the independent-samples *t*-test for normal data; categorical variables were compared using the *χ*^2^ test or Fisher’s exact test, as appropriate. Two-sided *p*-values <0.05 were considered statistically significant. Because all predictors were derived from CTA-based structural measurements (no subjective rating scales) and image quality was high, the analytic dataset contained no missing values; therefore, a complete-case analysis approach was used without imputation.

### 2.5 Regression analyses and model development

To explore structural risk factors for ICA contact, we fitted univariable and multivariable binary logistic regression models including sex, age, left/right SPL, IA, and FTA. All continuous predictors were *Z*-standardized prior to modeling; sex was dummy-coded with males as the reference. Variables with *p* < 0.10 in univariable analyses or strong *a priori* plausibility were entered into the multivariable model, with preference given to anatomically grounded measures such as sex and SPL. Given the limited number of candidate predictors (*n* = 5) and their theory-driven selection, automated procedures (e.g., LASSO or stepwise methods) were not used to mitigate overfitting and preserve interpretability. To compare alternative length metrics for predicting ICA contact—side-specific SPL, bilateral SPLs, bilateral mean SPL, and bilateral maximum SPL—we plotted receiver operating characteristic (ROC) curves, calculated the area under the curve (AUC), and identified the optimal cut-off using the Youden index, reporting sensitivity and specificity. The best-performing SPL metric was then combined with statistically significant covariates to construct a nomogram for ICA-contact risk prediction.

### 2.6 Internal validation, calibration, and clinical utility

Internal validation was performed using bootstrap resampling with 1,000 repetitions. Calibration was assessed by calibration plots comparing predicted probabilities with observed event rates. Clinical utility was evaluated using decision curve analysis (DCA) across clinically relevant risk-threshold ranges.

## 3 Results

### 3.1 Baseline characteristics and between-group comparisons

A total of 414 patients were included. Based on whether the styloid process was in direct contact with the ipsilateral internal carotid artery (ICA), participants were classified into a contact group (*n* = 110, 26.6%) and a non-contact group (*n* = 304, 73.4%). Baseline characteristics and styloid parameters are summarized in Table 1. The median age was 64.0 years [interquartile range (IQR), 55.0–71.0] in the contact group and 61.5 years (IQR, 54.0–69.0) in the non-contact group, with no significant difference in age distribution (*Z* = −1.251, *p* = 0.211). Sex distribution differed significantly (*χ*^2^ = 8.102, *p* = 0.004): the proportion of men was lower in the contact group than in the non-contact group (51.8% vs. 67.1%), suggesting that women were more likely to exhibit styloid–ICA contact as a potential structural susceptibility factor.

**Table 1:** Baseline characteristics and styloid process parameters by styloid–internal carotid artery (ICA) contact status.

Variable	Overall	Non-contact group	Contact group	Statistic	*p*-value
	*n* = 414 (100%)	*n* = 304 (73.4%)	*n* = 110 (26.6%)		
Age (years)	63.0 (54.0, 69.0)	61.5 (54.0, 69.0)	64.0 (55.0, 71.0)	−1.251	0.211^a^
Gender, *n* (%)				8.102	**0.004** ^b^
Male	261 (63.0%)	204 (67.1%)	57 (51.8%)		
Female	153 (37.0%)	100 (32.9%)	53 (48.2%)		
SPL (mm)					
Left	28.0 (24.0, 33.0)	26.9 (23.3, 31.2)	31.5 (27.4, 39.5)	−6.492	**<0.001** ^a^
Right	27.4 (23.3, 32.9)	26.2 (22.9, 30.5)	31.5 (26.9, 37.3)	−6.686	**<0.001** ^a^
IA (°)					
Left	19.0 (16.0, 23.0)	19.0 (16.0, 24.0)	19.5 (16.0, 23.0)	−0.084	0.933^a^
Right	22.0 (18.0, 26.0)	22.0 (18.0, 27.0)	22.0 (18.0, 26.0)	−0.226	0.821^a^
FTA (°)					
Left	27.0 (22.2, 31.0)	27.0 (23.0, 31.0)	26.0 (21.8, 31.0)	−0.810	0.418^a^
Right	28.0 (24.0, 33.0)	29.0 (25.0, 33.0)	28.0 (23.0, 32.3)	−1.099	0.272^a^

SPL, styloid process length; IA, medial inclination angle; FTA, anterior tilt angle. Continuous variables are presented as median (IQR) or mean ± SD where normally distributed; categorical variables as *n* (%). All tests are two-sided with *α* = 0.05; statistically significant *p*-values (<0.05) are shown in bold.

a Mann–Whitney *U* test.

b Pearson’s *χ*^2^ test.

With respect to styloid process length (SPL), bilateral SPLs were significantly longer in the contact group than in the non-contact group (both *p* < 0.001). Median (IQR) left-sided SPLs were 31.5 (27.4–39.5) mm in the contact group versus 26.9 (23.3–31.2) mm in the non-contact group (*Z* = −6.492). Median (IQR) right-sided SPLs were 31.5 (26.9–37.3) mm versus 26.2 (22.9–30.5) mm, respectively (*Z* = −6.686). These findings indicate that greater styloid length is closely associated with the occurrence of styloid–ICA contact. In contrast, there were no significant between-group differences in angular parameters—medial inclination angle (IA) or anterior tilt angle (FTA)—on either side (all *p* > 0.05), suggesting a limited influence of angular measures on ICA contact in this cohort.

### 3.2 Factors associated with styloid–ICA contact

**Figure 1:**
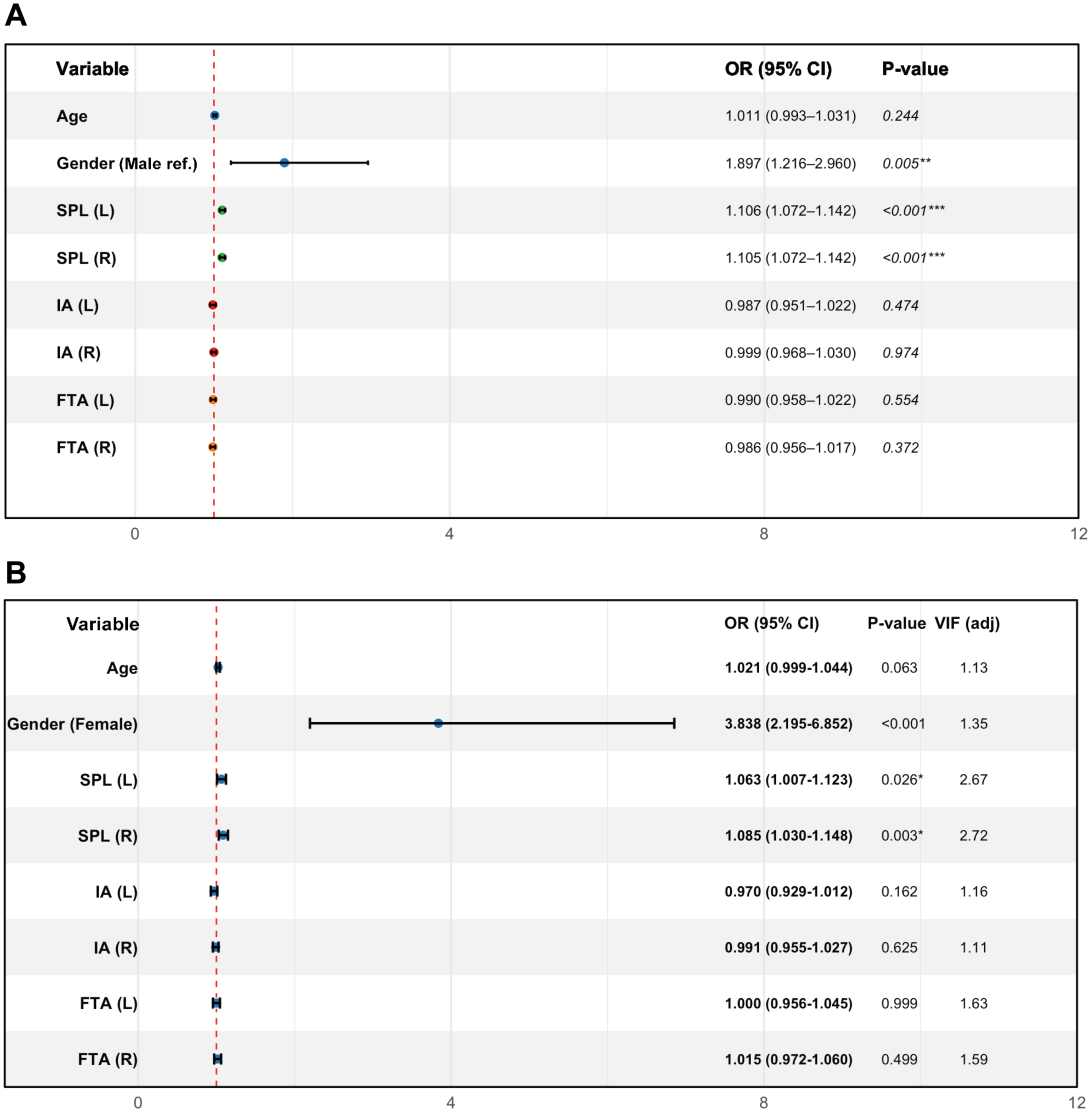
Forest plots of univariable and multivariable logistic regression for factors associated with internal carotid artery (ICA) contact. **(A)** Univariable logistic regression. **(B)** Multivariable logistic regression. Points represent odds ratios (ORs); horizontal bars indicate 95% confidence intervals (95% CIs). The vertical dashed line represents OR = 1.0 (no association). SPL-L/SPL-R, left/right styloid process length; IA-L/IA-R, left/right medial inclination angle; FTA-L/FTA-R, left/right anterior tilt angle; VIF (adjusted), variance inflation factor. Significance: *p* < 0.05 (*), *p* < 0.01 (**), and *p* < 0.001 (***).

Univariable logistic regression (Figure 1A) showed a significant association between sex and ICA contact (female vs. male: OR = 1.897, 95% CI 1.216–2.960, *p* = 0.005), indicating that women had 1.897 times the odds observed in men. Bilateral SPLs were positively associated with contact (both *p* < 0.001). For each 1-mm increase in SPL, the odds of left-sided contact increased (OR per 1 mm = 1.106, 95% CI 1.072–1.142), and the odds of right-sided contact increased (OR per 1 mm = 1.105, 95% CI 1.072–1.142). Age, bilateral IA, and FTA were not significantly associated with contact (all *p* > 0.05).

Multivariable logistic regression (Figure 1B) further demonstrated that, after adjustment for potential confounders (age, bilateral IA, and FTA), sex and bilateral SPL remained independent risk factors for ICA contact. Using men as the reference category (denoted “ref.” in the figure), women had 3.838 times the odds (OR = 3.838, 95% CI 2.176–6.771, *p* < 0.001). For each 1-mm increase in SPL, the odds of left- and right-sided contact increased (left SPL: OR = 1.063, 95% CI 1.007–1.122, *p* = 0.026; right SPL: OR = 1.085, 95% CI 1.028–1.145, *p* = 0.003). Age, bilateral IA, and FTA remained non-significant in the multivariable model (all *p* > 0.05). Taken together, sex and SPL emerged as the principal structural predictors of ICA contact, whereas angular parameters contributed little to contact risk in this dataset.

### 3.3 Sex-stratified comparison of structural parameters and association with styloid–ICA contact

Given that sex emerged as an independent predictor in the overall analysis, we conducted sex-stratified comparisons of structural parameters. Among the 414 patients, 261 were men (63.0%) and 153 were women (37.0%). There was no between-sex difference in age distribution (median 63.0 vs. 63.0 years; *Z* = −0.849, *p* = 0.396). Styloid-related parameters differed significantly by sex (Table 2): men had greater bilateral styloid process lengths (SPLs) (both *p* < 0.001) and larger anterior tilt angles (FTAs) (both *p* < 0.001), whereas women had larger medial inclination angles (IAs) (left IA: 21.0° vs. 19.0°, *Z* = −3.443, *p* < 0.001; right IA: 23.0° vs. 22.0°, *Z* = −2.585, *p* = 0.010). These findings suggest that, although men exhibit longer styloids and greater anterior tilt, women—potentially due to a relatively narrower cranial base–ICA corridor—may be more prone to styloid–ICA contact under comparable length conditions, a sex-specific structural susceptibility that may modulate the pathogenesis of ES–CA.

**Table 2:** Sex-stratified comparison of baseline characteristics and styloid process parameters.

Variable	Male	Female	Statistic	*p*-value
	*n* = 261 (63.0%)	*n* = 153 (37.0%)		
Age (years)	63.00 (54.0 69.0)	63.00 (55.0, 70.0)	−0.849	0.396
SPL (mm)				
Left	28.80 (25.1, 34.0)	26.10 (22.5, 31.2)	−4.267	**<0.001**
Right	28.10 (24.1, 33.5)	25.00 (22.4, 30.3)	−5.930	**<0.001**
IA (°)				
Left	19.00 (15.00, 23.0)	21.00 (17.00, 24.00)	−3.443	**<0.001**
Right	22.00 (18.00, 25.00)	23.00 (20.00, 27.00)	−2.585	**0.010**
FTA (°)				
Left	28.00 (24.00, 32.00)	25.00 (21.00, 29.00)	−3.977	**<0.001**
Right	30.00 (26.00, 35.00)	26.00 (21.00, 30.00)	−4.068	**<0.001**

Data are presented as median (interquartile range, IQR). SPL, styloid process length; IA, medial inclination angle; FTA, anterior tilt angle. Between-sex comparisons used the Mann–Whitney *U* test (*Z* statistic). All tests are two-sided with *α* = 0.05; statistically significant *p*-values (<0.05) are shown in bold.

### 3.4 Sex-specific analyses of factors associated with styloid–ICA contact

#### 3.4.1 Within-sex comparisons of styloid parameters between contact and non-contact groups

**Figure 2:**
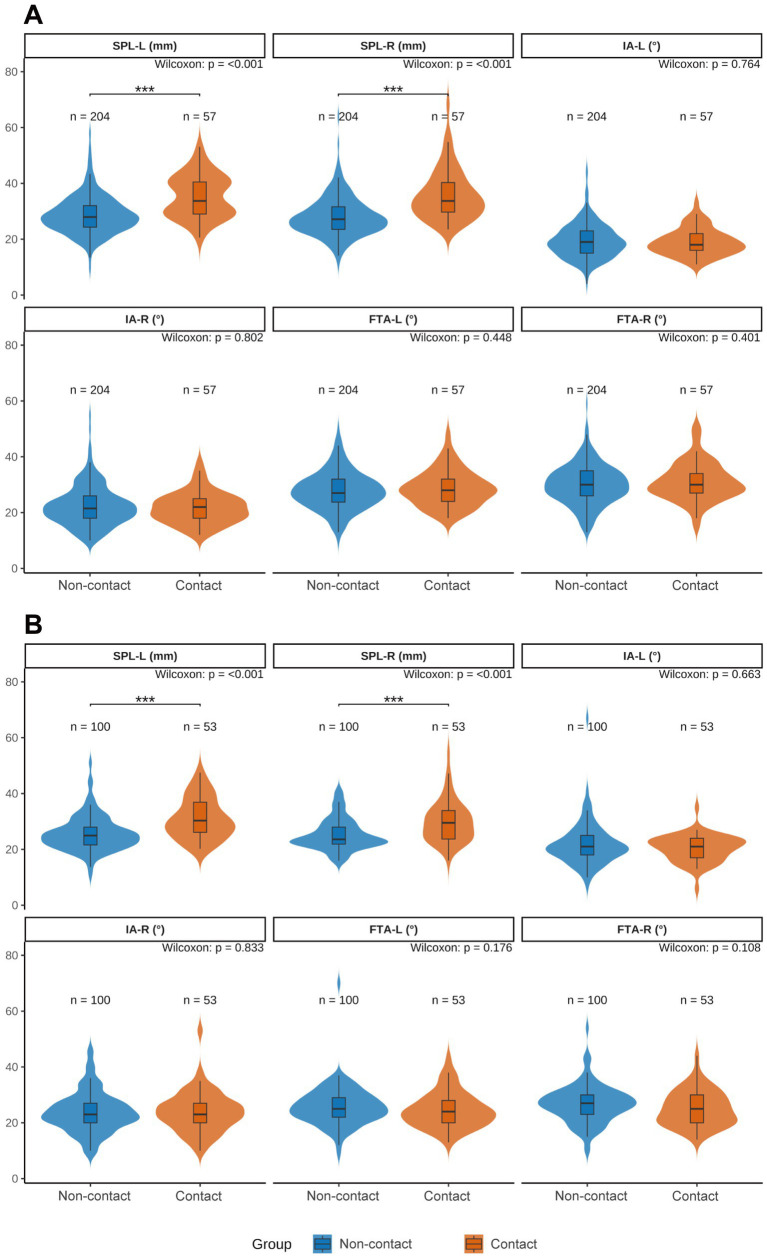
Sex-stratified violin plots comparing styloid process parameters between carotid-contact and non-contact groups. **(A)** Men; **(B)** women. SPL-L, left styloid process length; SPL-R, right styloid process length; IA-L/IA-R, left/right medial inclination angle; FTA-L/FTA-R, left/right anterior tilt angle. Blue indicates the non-contact group, orange indicates the contact group. Between-group differences were assessed with the Wilcoxon rank-sum test (Mann–Whitney *U*). Significance: *p* < 0.05 (*), *p* < 0.01 (**), and *p* < 0.001 (***).

To delineate sex-specific associations between contact status and styloid metrics, we compared styloid parameters between the contact and non-contact groups within each sex. As illustrated in Figure 2, both sexes showed a consistent pattern: bilateral SPLs were significantly longer in the contact group than in the non-contact group (both *p* < 0.001), whereas bilateral IAs and FTAs did not differ significantly between groups (all *p* > 0.05).

#### 3.4.2 Univariable logistic regression within sex subgroups

Building on the distributional comparisons, we quantified the association of each parameter with styloid–ICA contact using univariable logistic regression. As shown in Supplementary Figure S1, bilateral SPLs were positively and significantly associated with contact in both sexes (all *p* < 0.001): Men—left SPL: OR = 1.126, 95% CI 1.080–1.178; right SPL: OR = 1.129, 95% CI 1.084–1.182. Women—left SPL: OR = 1.121, 95% CI 1.064–1.189; right SPL: OR = 1.120, 95% CI 1.059–1.190. Age, bilateral IA, and FTA were not significantly associated with contact in either sex (all *p* > 0.05).

#### 3.4.3 Multivariable logistic regression within sex subgroups

To control for potential confounding by age, bilateral IA, and bilateral FTA, we fitted multivariable logistic models separately for each sex (Supplementary Figure S2). Men (Supplementary Figure S2A): right-sided SPL remained significantly and positively associated with styloid–ICA contact (OR = 1.101, 95% CI 1.032–1.184, *p* = 0.006), whereas left-sided SPL was not significant (*p* = 0.179). Women (Supplementary Figure S2B): left-sided SPL remained significantly and positively associated with contact (OR = 1.092, 95% CI 1.002–1.198, *p* = 0.050), whereas right-sided SPL was not significant (*p* = 0.286). Across all sex-specific multivariable models, variance inflation factors (VIFs) were <3, indicating no meaningful multicollinearity. In summary, increasing SPL was associated with greater odds of styloid–ICA contact. In men, a longer right-sided SPL constituted an independent predictor (each 1-mm increase associated with ~10.1% higher odds); in women, a longer left-sided SPL was independently associated with contact (each 1-mm increase associated with ~9.2% higher odds). Age, IA, and FTA showed no independent associations in either sex.

### 3.5 Receiver operating characteristic analysis of styloid-length metrics for predicting styloid–ICA contact and selection of the optimal predictor

Given that SPL was a key correlate across subgroups—and that the associations between left/right SPL and styloid–ICA contact differed by sex—we compared the diagnostic performance of four SPL metrics to identify the optimal predictor. Specifically, we evaluated: (1) bilateral SPLs (*n* = 828), treating the left and right styloids from all 414 patients as independent observations and analyzing them jointly; (2) side-specific (ipsilateral) SPLs (*n* = 414), analyzing left and right SPLs separately at the patient level; (3) bilateral mean SPL (*n* = 414), defined as [(left SPL + right SPL)/2] per patient; and (4) bilateral maximum SPL (*n* = 414), defined as max (left SPL, right SPL) per patient. All metrics were assessed by ROC analysis in the overall cohort and within sex strata.

**Figure 3:**
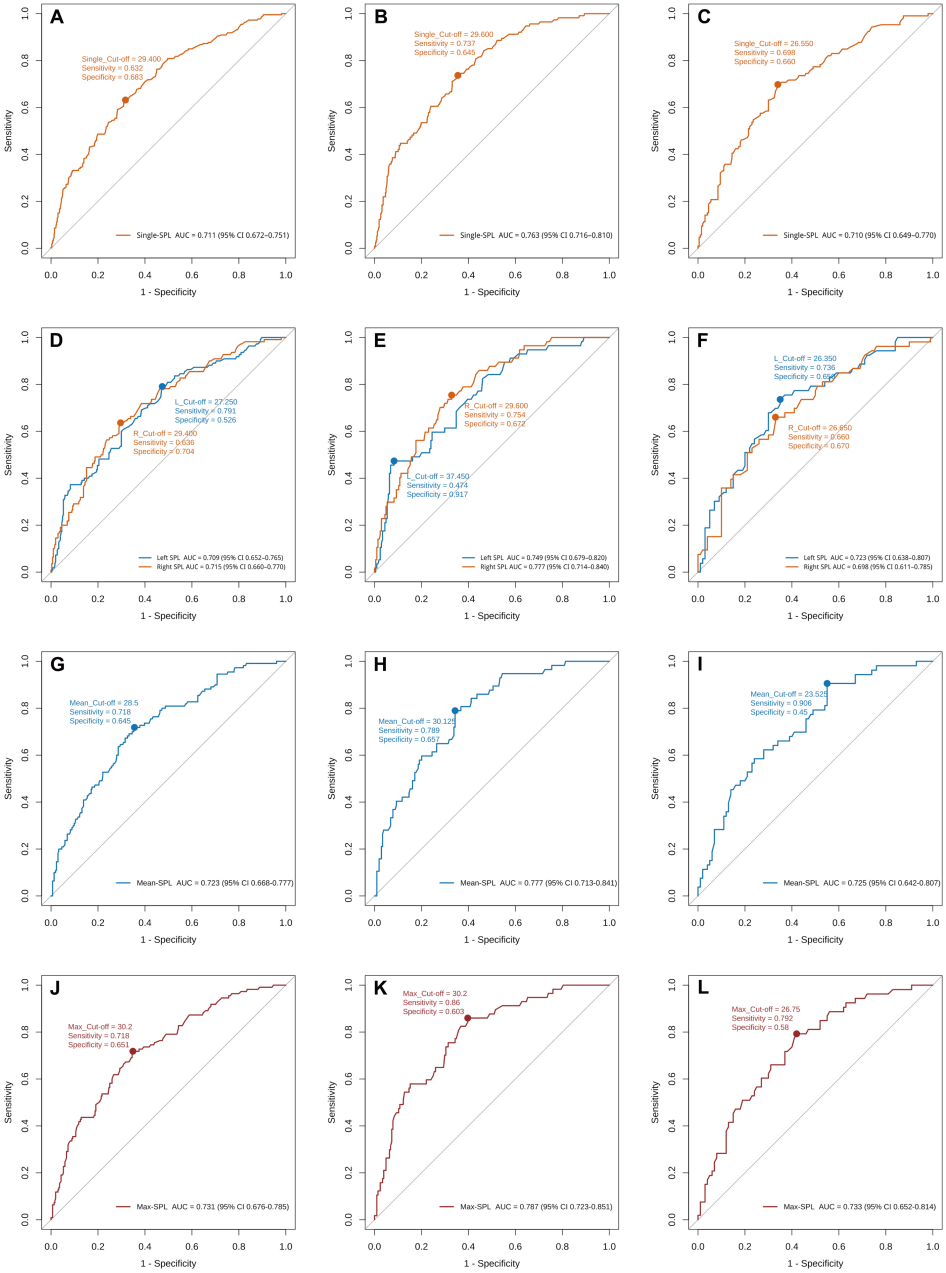
ROC curves of styloid-length metrics for predicting internal carotid artery (ICA) contact in the overall cohort and by sex. **(A–C)** Bilateral styloid process lengths (SPLs) treated as independent observations (*n* = 828) in the overall cohort, men, and women, respectively. **(D–F)** Side-specific (ipsilateral) SPLs with left and right analyzed separately. **(G–I)** Bilateral mean SPL for each patient, defined as (left SPL + right SPL)/2. **(J–L)** Bilateral maximum SPL for each patient, defined as max (left SPL, right SPL). For each panel, the area under the curve (AUC), the optimal cut-off (Youden index), sensitivity, and specificity are displayed on the plot. SPL, styloid process length.

ROC analyses (Figure 3) showed measurable diagnostic value for all metrics, with clear sex-specific differences in cut-off values. Notably, the bilateral maximum SPL provided the best performance in the overall cohort and in both sex strata. Results were as follows: overall, AUC = 0.731 (95% CI, 0.676–0.785), cut-off = 30.20 mm, sensitivity = 0.718, specificity = 0.651; in men, AUC = 0.787 (95% CI, 0.723–0.851), cut-off = 30.20 mm, sensitivity = 0.860, specificity = 0.603; in women, AUC = 0.733 (95% CI, 0.652–0.814), cut-off = 26.75 mm, sensitivity = 0.792, specificity = 0.580. Importantly, the sex-specific cut-offs for the bilateral maximum SPL differed by 3.45 mm (30.20 mm in men vs. 26.75 mm in women), further supporting the sex-specific nature of the SPL–contact relationship. On the basis of its superior predictive performance, the bilateral maximum SPL was selected as the core metric for model development.

### 3.6 Nomogram development and validation

**Figure 4:**
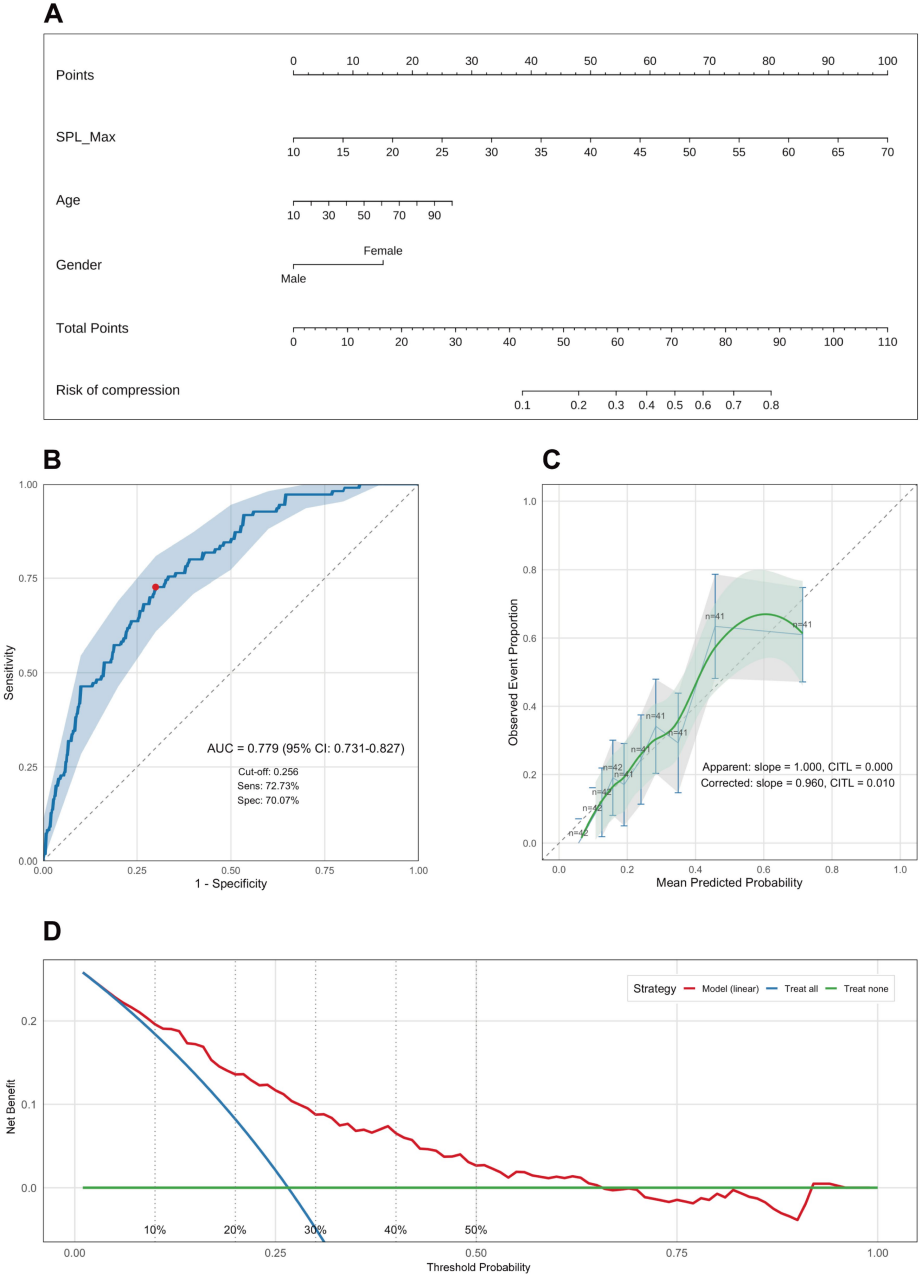
Nomogram for predicting internal carotid artery (ICA) contact and model performance evaluation. **(A)** Nomogram constructed using bilateral maximum styloid process length (SPL-Max), age, and sex (plotted as Gender). The total score for a given patient maps to the predicted probability of internal carotid artery (ICA) contact. **(B)** Receiver operating characteristic (ROC) curve demonstrating model discrimination: AUC = 0.779 (95% CI: 0.731–0.827); optimal cut-off by Youden index = 0.296; sensitivity = 72.73%, specificity = 70.07%. **(C)** Calibration plot comparing predicted probabilities with observed rates. Apparent calibration: slope = 1.000, calibration-in-the-large (CITL) = 0.000. Bootstrap-corrected: slope = 0.960, CITL = 0.010. **(D)** Decision curve analysis (DCA) shows that the model achieves a positive net benefit across the 1–65% threshold range, supporting its potential clinical utility. SPL-Max, bilateral maximum styloid process length; AUC, area under the curve; CI, confidence interval; CITL, calibration-in-the-large; DCA, decision curve analysis.

Building on the multivariable regression findings, and using the selected metric—the bilateral maximum styloid process length (SPL-Max)—together with sex and age, we developed a nomogram to estimate the probability of styloid–ICA contact (Figure 4A). Discrimination: the area under the receiver operating characteristic curve (AUC) was 0.779 (95% CI, 0.731–0.827), which falls within the 0.7–0.9 range and indicates good discriminative ability. The optimal probability threshold was 0.256, at which the sensitivity was 72.73% and the specificity was 70.07%, suggesting effective identification of individuals at higher versus lower risk of styloid–ICA contact (Figure 4B).

Predictive accuracy: the Brier score was 0.160, indicating a reasonable prediction error. After 1,000-bootstrap internal validation, the optimism-corrected AUC was 0.770 and the optimism-corrected Brier score was 0.165, reflecting only modest attenuation and overall stability of model performance. Calibration: following 1,000-bootstrap correction, the calibration slope was 0.960, close to 1.0, demonstrating good agreement between predicted probabilities and observed outcomes (Figure 4C). Clinical utility: decision curve analysis showed that, across threshold probabilities of 1–65%, the model provided greater net benefit than either the “treat-all” or “treat-none” strategies, with particularly pronounced benefit at low-to-intermediate thresholds (0.01–0.50) (Figure 4D).

In summary, the proposed nomogram demonstrated good discrimination, calibration, and clinical utility, and offers a convenient, reliable quantitative tool for clinicians to assess the probability of styloid–ICA contact, supporting early structural ES–CA screening and risk stratification.

The complete model specification—including the logistic regression formula, coefficients, odds ratios, and 95% confidence intervals—is provided in Supplementary Table S1 to facilitate implementation and reproducibility.

## 4 Discussion

Drawing on 414 CTA-based three-dimensional reconstructions, we systematically evaluated the spatial relationship between the styloid process and the internal carotid artery (ICA) and developed a structural risk-prediction model. The principal findings were as follows: (1) the prevalence of *styloid–ICA contact* in the overall cohort was 26.6%; (2) age and angular parameters (anterior tilt angle, medial inclination angle) were not associated with contact; (3) women had substantially higher odds of contact than men and sex remained an independent predictor in multivariable analysis (OR = 3.838, 95% CI 2.176–6.771, *p* < 0.001); (4) the bilateral maximum styloid process length (max SPL) yielded the best discriminative performance (overall AUC = 0.731; men AUC = 0.787; women AUC = 0.733) with sex-specific optimal cut-offs (men 30.20 mm; women 26.75 mm); (5) the side of contact exhibited a sex-dependent bias (men, right-sided predominance; women, left-sided predominance); and (6) a nomogram integrating sex, age, and max SPL showed good discrimination, calibration, and clinical utility, with a stable net benefit across 1–65% decision thresholds on DCA. Collectively, these results suggest that, for the carotid variant of ES–CA, a “length-dominant, sex-modulated” structural model aligns more closely with the underlying pathophysiology.

**Figure 5:**
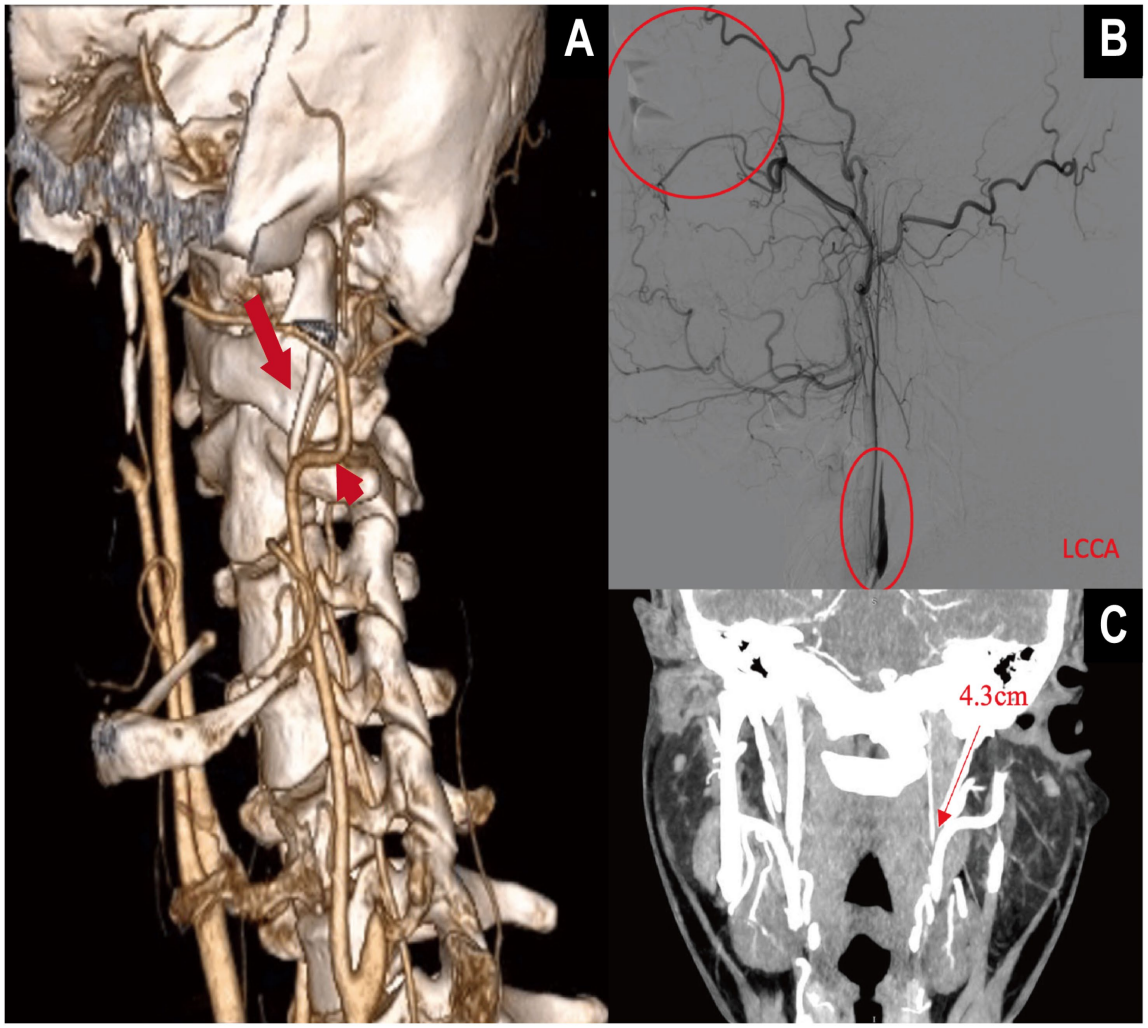
Multimodal imaging demonstration of internal carotid artery (ICA) dissection secondary to elongated styloid process in vascular Eagle syndrome (VES). **(A)** Three-dimensional CTA with volume rendering (VR) shows a markedly elongated styloid process. The long straight arrow indicates the elongated styloid process, while the short straight arrow marks the contact point with the ipsilateral internal carotid artery (ICA). **(B)** Digital subtraction angiography (DSA) reveals a “rat-tail” tapering deformity of the ICA, consistent with arterial dissection, without collateral reperfusion. The patient presented with central retinal artery occlusion in the left eye. **(C)** The VR image shows the left styloid process measuring 4.3 cm, located adjacent to the ICA, supporting the anatomical basis of compression.

Prior work commonly used SPL >3.0 cm as an empirical threshold for elongation (7), and defined angular abnormalities using IA >15° and FTA >30° (9). However, radiographic elongation is not sufficient for ES–CA. In our series, only six patients (1.4% of the cohort; 5.4% among contact-positive cases) were diagnosed with ES–CA–related ICA dissection or stenosis leading to ischemic stroke, a clinically uncommon but easily underrecognized entity (Figure 5). This underscores the need for proactive structural risk identification. Our nomogram is expressly intended to support quantitative risk assessment in outpatient or imaging settings before overt stroke, thereby facilitating earlier recognition and individualized intervention to prevent irreversible ischemic events.

With respect to structural determinants, our data support a “length-dominant” mechanism: the odds of *styloid–ICA contact* were independent of age but strongly associated with sex, with women at higher risk and sex remaining an independent predictor (OR = 3.838). Although female predominance has been reported in SPS overall (10, 13), robust quantification of sex-specific structural risk within ES–CA has been limited. A plausible explanation is that women may have a narrower cranial base–ICA corridor and differences in soft-tissue compliance, rendering contact more likely at comparable lengths; this hypothesis warrants testing with dynamic imaging and anatomic studies.

Further analysis showed that max SPL provided the highest discriminative ability across the full cohort and within sex strata (AUC = 0.731–0.787), with sex-specific cut-offs differing by 3.45 mm (30.20 mm in men vs. 26.75 mm in women). These findings argue against a single universal threshold and support sex-stratified cut-offs for individualized risk appraisal. To our knowledge, ES–CA–specific stratified thresholds have not been rigorously established previously; our data offer an initial, clinically actionable reference point.

In parallel, we systematically evaluated angular parameters. Despite men exhibiting longer SPLs and larger FTAs (and slightly smaller IAs) than women, IA and FTA were not independent predictors in multivariable models. This diverges from some studies focusing on symptomatic ES. For example, Duan et al. (17) proposed an “R-value” (length × angle × cranial morphology) that discriminated symptomatic ES (AUC ≈ 0.86 left, 0.82 right). The apparent discrepancy likely reflects different endpoints: their outcome was clinical symptomatology, whereas ours was a structural risk state (*styloid–ICA contact*). Symptoms may emerge from multifactorial interactions, while static contact formation is more directly governed by “reachable distance” (i.e., length).

Consistent with our inferences, a systematic review of 56 ES–CA cases highlighted direct mechanical compression as a principal mechanism and noted that hemodynamic fragility (e.g., incomplete circle of Willis, limited perfusion reserve) may amplify structural effects (18). Conversely, radiographic elongation alone is not equivalent to clinical disease. In a cohort of 3,962 individuals, the prevalence of ESP was 4.5% while symptoms were far less frequent; local tissue changes (e.g., post-tonsillectomy scarring) may modulate symptom expression (19). This aligns with our observation that *styloid–ICA contact* is more common than stroke events and reinforces that risk prediction should rely on precise imaging markers rather than length alone.

From a translational standpoint, Kumar et al. (20) developed an AI model on orthopantomograms (OPG) for automatic detection of styloid elongation with near-ideal AUC, supporting a “AI prescreen → CTA confirmation → nomogram-based quantification” pathway. Our sex-specific laterality signal (men, right; women, left) may relate to individual cranio-cervical anatomy, positional habits (e.g., preferred sleeping side) (21), or flow patterns; prospective evaluation using 4D-CTA or dynamic ultrasound is warranted. In light of current evidence, we suggest three practice points for ES–CA: (1) adopt sex-stratified SPL cut-offs (men ≥30.20 mm; women ≥26.75 mm) to improve risk identification; (2) account for side predilection in pre-operative planning and follow-up; and (3) explore the AI-prescreen → CTA → nomogram workflow to enhance detection efficiency and individualized management.

### 4.1 Innovation

We defined *styloid–ICA contact* as a structural endpoint, proposed and validated sex-specific SPL thresholds, and constructed a pragmatic, visually interpretable nomogram. DCA demonstrated stable net benefit across 1–65% thresholds, supporting its potential utility for early screening and precision intervention.

### 4.2 Therapeutic implications

Modern ES–CA management spans conservative measures (analgesia, posture adjustment), endovascular options (e.g., stenting in selected dissection/stenosis), and definitive decompression (styloidectomy). In selected stroke cases driven by ICA impingement/variant anatomy, styloidectomy alone has relieved symptoms and prevented recurrence (22), underscoring the complementary roles of structural decompression and individualized vascular therapy.

### 4.3 Limitations

(1) Single-center retrospective design with potential selection bias limits generalizability; (2) angular measurements may vary with CTA reconstruction planes and patient positioning—despite demonstrated inter-rater agreement, minor error is unavoidable; (3) the limited, theory-driven predictor set precluded use of automated selection (e.g., LASSO) to avoid overfitting and preserve interpretability; (4) the imaging-database design did not capture systematic symptom or outcome data, preventing longitudinal linkage between contact and stroke risk. Future work should employ multicenter prospective cohorts with symptom tracking, stroke outcomes, dynamic flow imaging, and AI-assisted analytics, and evaluate interventions (e.g., posture strategies, flow monitoring, early surgery) for stroke prevention.

All model coefficients, odds ratios, and the full prediction equation are presented in Supplementary Table S1 to support clinical implementation and reproducibility.

In conclusion, SPL is the central morphologic marker for ES–CA structural risk. We introduce sex-specific predictive thresholds and a quantitative nomogram that operationalize the intermediate step in the continuum “anatomic elongation → structural contact → ischemic events,” supporting earlier identification and precision management at subclinical stages.

## Data Availability

The raw data supporting the conclusions of this article will be made available by the authors, without undue reservation.

## Supplementary material

SUPPLEMENTARY FIGURE S1 Sex-stratified forest plots of univariable logistic regression for factors associated with internal carotid artery (ICA) contact. **(A)** Men; **(B)** women. OR, odds ratio; 95% CI, 95% confidence interval; SPL-L/SPL-R, left/right styloid process length; IA-L/IA-R, left/right medial inclination angle; FTA-L/FTA-R, left/right anterior tilt angle. The vertical dashed line denotes OR = 1.0 (no association). L, left; R, right. Significance: *p* < 0.05 (*), *p* < 0.01 (**), and *p* < 0.001 (***).

SUPPLEMENTARY FIGURE S2 Sex-stratified forest plots of multivariable logistic regression for factors associated with internal carotid artery (ICA) contact. **(A)** Men; **(B)** women. OR, odds ratio; 95% CI, 95% confidence interval; VIF (adj), adjusted variance inflation factor; SPL-L/SPL-R, left/right styloid process length; IA-L/IA-R, left/right medial inclination angle; FTA-L/FTA-R, left/right anterior tilt angle. The vertical dashed line denotes OR = 1.0 (no association). L, left; R, right. Significance: *p* < 0.05 (*), *p* < 0.01 (**), and *p* < 0.001 (***).

## Glossary

AUC: Area under the receiver operating characteristic curve
CAD: Carotid artery dissection (in this article; not coronary artery disease)
CI: Confidence interval
CITL: Calibration-in-the-large
CT: Computed tomography
CTA: Computed tomography angiography
DCA: Decision curve analysis
DSA: Digital subtraction angiography
ESP: Elongated styloid process
FTA: Anterior tilt angle (also called forward tilt angle)
IA: Medial inclination angle
ICA: Internal carotid artery
IQR: Interquartile range
MCA: Middle cerebral artery
MIP: Maximum intensity projection
OR: Odds ratio
ROC: Receiver operating characteristic
SD: Standard deviation
SP: Styloid process
SPL: Styloid process length
SPL-L/SPL-R: Left/Right styloid process length
SPL-Max: Maximum bilateral styloid process length (per patient, the larger of left/right SPL)
SPS: Styloid process syndrome (Eagle syndrome)
TCD: Transcranial Doppler
VES: Vascular Eagle syndrome
VIF: Variance inflation factor
VIF (adj): Adjusted variance inflation factor
VR: Volume rendering
L/R: Left/Right
