# Supplementary figures and images for: CTA-based risk assessment of the carotid variant of Eagle syndrome: development and internal validation of a nomogram
Source: Front Neurol. 2025 Oct 30;16:1699139. doi: 10.3389/fneur.2025.1699139 (PMC12611689; doi:10.3389/fneur.2025.1699139)

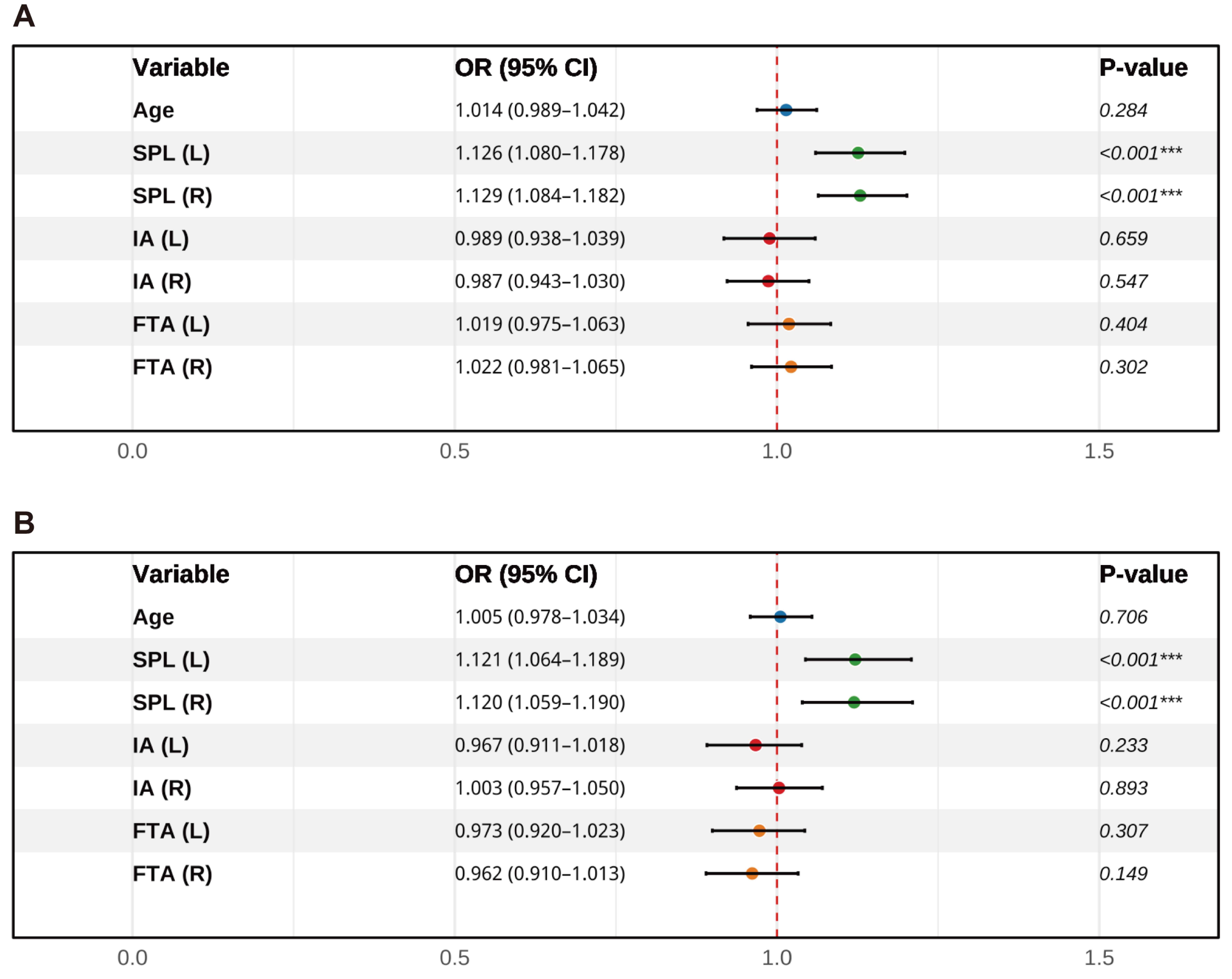

Supplement: Supplementary file 1 [file Image_1.jpeg]

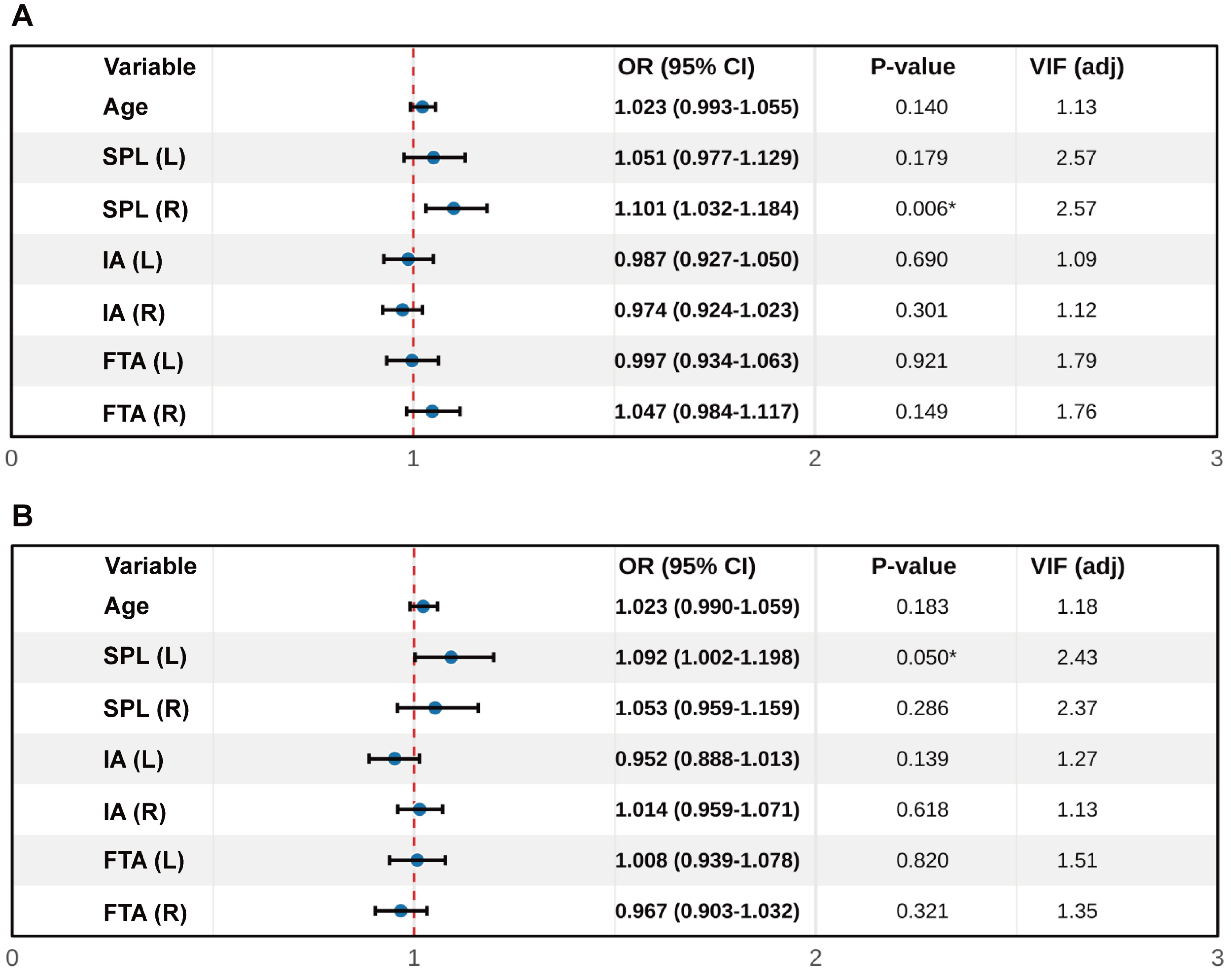

Supplement: Supplementary file 2 [file Image_2.jpeg]
