# Supplementary material for: CTA-based risk assessment of the carotid variant of Eagle syndrome: development and internal validation of a nomogram
Source: Front Neurol. 2025 Oct 30;16:1699139. doi: 10.3389/fneur.2025.1699139 (PMC12611689; doi:10.3389/fneur.2025.1699139)
Supplement: Supplementary file 3 [file Table_1.docx]

Supplementary Material

# Supplementary Table S1. Full Specification of the Final Logistic Regression Model

Model type: Binary logistic regression

Outcome: Presence of ICA contact (1 = contact, 0 = non-contact)

Predictors: SPL_max (in mm), Age (in years), Gender (reference = Female)

Equation (logit form):

logit(p) = -5.851 + 0.130 × SPL_max + 0.023 × Age - 1.174 × Gender[Male]

Where Gender[Male] is a binary indicator: 1 for male, 0 for female.

Predicted probability:

p = exp(logit) / (1 + exp(logit))

Regression coefficients, Odds Ratios (ORs), and 95% Confidence Intervals:

| Predictor | β (Coefficient) | Standard Error | Odds Ratio (OR) | 95% CI (OR) |
| --- | --- | --- | --- | --- |
| Intercept | -5.851 | 0.931 | — | — |
| SPL_max (per mm) | 0.130 | 0.018 | 1.139 | 1.099 – 1.179 |
| Age (per year) | 0.023 | 0.011 | 1.023 | 1.002 – 1.045 |
| Gender (Male) | -1.174 | 0.265 | 0.309 | 0.184 – 0.520 |
